# Hepatitis C virus (HCV) infection in Malaysia: findings from a nationwide cross-sectional study

**DOI:** 10.1016/j.lanwpc.2023.100802

**Published:** 2023-05-25

**Authors:** Eida Nurhadzira Muhammad, Mohd Hatta Abdul Mutalip, Zhuo-Lin Chong, Huan-Keat Chan, Fazidah Yuswan, Noor Ani Ahmad, Muhammad Radzi Abu Hassan

**Affiliations:** a Institute for Public Health, National Institute of Health, Setia Alam, Selangor, Malaysia; b Deputy Director General's (Research & Technical Support) Office, Ministry of Health Malaysia, Putrajaya, Malaysia; c HIV, STIs and Hepatitis C Control Sector, Disease Control Division, Ministry of Health Malaysia, Putrajaya, Malaysia

New cases of HCV are reported each year, with an estimated 58 million people worldwide,^1^ and 10 million people in the Western Pacific, carrying the infection.^2^ While in Malaysia, McDonald et al. previously modelled that, 2.5% prevalence with an estimated 453,700 HCV infection.^3^ Although models might provide insightful information, it might take some time to interpret the findings. Hence, seroprevalence study was conducted to estimate the true burden of HCV infection and chronic HCV in Malaysia and to determine the proportion of diagnosis and treatment status among those who were infected with HCV.

We organized a cross-sectional study at the end of 2020 across Malaysia. We used a two-stage stratified random sampling technique to select representative community dwelling individuals aged ≥15 years. The optimum size sample required was 5000 based on the single proportion sample size formula, adjusted for design effect and non-response. We obtained written consents and assent (if ≤ 18 years) and conducted face-to-face interviews to collect sociodemographic characteristics and HCV status. We collected blood samples and extracted serum to test for HCV antibodies. A positive sample (denotes HCV infection) would be tested further for HCV core antigen. Positive results on both tests denote chronic infection.^4^ We used SPSS v26 to perform descriptive analysis and apply weights in complex sampling analysis to obtain representative prevalence's and their 95% Confidence Interval (CI) (Detailed methodology and other information in Supplementary Materials).

Out of 5957 eligible individuals, test results were available for 4076 (response rate 68.4%). Their mean age was 41.2 ± 17.3 years. The weighted prevalence of HCV infection in Malaysia was 0.4% (95% CI 0.2–0.7) amounting to an estimated of 90,119 infected population age ≥15 years. More than half of them (≈51,675) chronically infected with the prevalence of 0.2% (95% CI 0.1–0.4) (Table 1). All participants tested positive in our study were previously undiagnosed, hence received referral for further management.

**Table 1:** Sociodemographic and seroprevalence of HCV infection among participants age 15 years old and above in Malaysia.

Variables	Participant^a^, n (%)	Estimated population^a^, N (%)	HCV Infection				Chronic HCV Infection			
			n	Estimated population, N	Prevalence^b^ (%)	RSE	n	Estimated population, N	Prevalence^b^ (%)	RSE
**Overall**	4076 (100.0)	24,142,146 (100.0)	19	90,119	0.4 (0.2, 0.7)	0.331	14	51,675	0.2 (0.1, 0.4)	0.346
**Residential area**										
Urban	2186 (53.6)	18,706,908 (77.5)	9	71,398	0.4 (0.2, 0.9)	0.408^c^	5	34,977	0.2 (0.1, 0.5)	0.479^c^
Rural	1890 (46.4)	5,435,238 (22.5)	10	18,720	0.3 (0.2, 0.7)	0.349	9	16,698	0.3 (0.1, 0.6)	0.374^c^
**Gender**										
Male	1919 (47.1)	12,430,875 (51.5)	14	82,609	0.7 (0.3, 1.3)	0.358	9	44,164	0.4 (0.2, 0.8)	0.396^c^
Female	2157 (52.9)	11,711,271 (48.5)	5	7510	0.1 (0.0, 0.2)	0.512^c^	5	7511	0.1 (0.0, 0.2)	0.512^c^
**Age group**										
15–39 years	2040 (50.1)	14,090,749 (58.4)	5	28,007	0.2 (0.1, 0.6)	0.533^c^	5	28,007	0.2 (0.1, 0.6)	0.533^c^
40–59 years	1310 (32.1)	6,650,625 (27.5)	10	39,995	0.6 (0.3, 1.4)	0.411^c^	7	20,079	0.3 (0.1, 0.8)	0.496^c^
60 years and above	726 (17.8)	3,400,772 (14.1)	4	22,117	0.7 (0.2, 2.5)	0.694^c^	2	3589	0.1 (0.0, 0.5)	0.763^c^
**Ethnicity**										
Malay	2492 (61.1)	11,545,690 (47.8)	13	46,660	0.4 (0.2, 0.8)	0.334	9	22,846	0.2 (0.1, 0.5)	0.435^c^
Chinese	387 (9.5)	5,449,457 (22.6)	1	14,631	0.3 (0.0, 2.0)	1.030^c^	–	–	–	–
Indian	200 (4.9)	1,526,953 (6.3)	1	6597	0.4 (0.1, 3.3)	1.035^c^	1	6597	0.4 (0.1, 3.3)	1.035^c^
Bumiputera Sabah	704 (17.3))	2,693,310 (11.2)	4	22,232	0.8 (0.2, 2.8)	0.614^c^	4	22,232	0.8 (0.2, 2.8)	0.614^c^
Others	293 (7.2)	2,926,737 (12.1)	–	–	–	–	–	–	–	–
**Marital status**										
Single	1164 (28.6)	7,639,678 (31.6)	5	76,607	0.4 (0.1, 1.0)	0.522^c^	2	7690	0.1 (0.0, 0.6)	0.872^c^
Married/Living with partner	2560 (62.8)	14,529,839 (60.2)	11	51,973	0.4 (0.1, 1.0)	0.496^c^	10	37,342	0.3 (0.1, 0.6)	0.422^c^
Widowed (er)/Divorcee	351 (8.6)	1,969,974 (8.2)	3	10,540	0.5 (0.2, 1.7)	0.593^c^	2	6643	0.3 (0.1, 1.4)	0.736^c^
**Highest education level**										
No formal education	235 (5.8)	1,319,923 (5.5)	1	2519	0.2 (0.0, 1.5)	1.042^c^	1	2519	0.2 (0.0, 1.5)	1.042^c^
Primary education	882 (21.8)	4,761,056 (20.0)	4	18,037	0.4 (0.1, 1.4)	0.671^c^	3	6536	0.1 (0.0, 0.5)	0.634^c^
Secondary education	1925 (47.6)	10,596,604 (44.5)	13	54,932	0.5 (0.3, 1.0)	0.327	10	42,620	0.4 (0.2, 0.9)	0.392^c^
Tertiary education	1005 (24.8)	7,158,253 (30.0)	0	–	–	–	0	–	–	–

a The number of participants in the study and representativeness of Malaysia's general population after applying weight.

b Denominator for each weighted prevalence was the total estimated population correspond subpopulation ≥15 years in Malaysia.

c The relative standard error (RSE) value more than 0.35 indicating a lack of precision. The point estimates should be interpreted strictly with its 95% confidence intervals.

Our HCV infection finding corroborates with population cohort studies (0.3%)^5^ but lower compared to study from McDonald et al.^3^ The prevalence is comparable to those in neighbouring countries like Indonesia (0.5%), and Thailand (0.5%) but lower than Vietnam (0.9%) and Cambodia (1.1%).^6^ Our study is the first to report chronic HCV infection in this region.

Our study found a lower burden of HCV and chronic HCV infections among the general population, but all of them we undiagnosed. In 2019, Malaysia released the National Strategic Plan for Hepatitis B and C 2019–2030 to combat hepatitis and one of the targets was to diagnose 90% of population living with viral hepatitis.^7^ Therefore, the Malaysian government took many initiatives such as conducting early screening to save more lives and prevent chronic sequelae that can burden the healthcare system. This early screening also included in the program among antenatal mothers as part of the prevention of mother-to-child transmission program. In addition, strengthening knowledge and awareness about HCV is important to curb the spread of the disease in the community. Having sufficient knowledge or awareness about HCV and its risk factors will foster early HCV self-screening, especially among those who engage in high-risk behaviours. Efforts to minimize the stigma associated with HCV testing have also been shown to encourage testing for early treatment. Data received from the Ministry of Health Malaysia reported, there were 3438 new chronic infection in 2019 and the number increased to 4626 in 2022 with a notification rate of 10.55 and 14.17 per 100,000 population, respectively.

Started year 2018, sofosbuvir and daclatasvir were used as the standard Direct-Acting Antivirals (DAAs) regimen to treat HCV infection in Malaysia, which is the choice of DAAs and length of treatment vary according to the stage of liver disease.^4^ Currently, the Ministry of Health was providing HCV screening and treatment in 63 public hospitals and 618 public health clinics across country, which have treated 4819 patients in 2022. Although treatment is free at public health facilities in Malaysia, there remain several barriers that patients may overcome while seeking treatment, such as the expense of transportation costs and loss productivity in jobs and daily activities because of multiple treatment follow-up.^8^

However, the prevalence of HCV infection is likely higher on the targeted populations e.g. prisoners or vulnerable population, which not covered in this study. Further studies and screening efforts must be concentrated towards these populations to high the yield and the rate. Failing to recognise them prevents linkage to care and effective HCV control. HCV screening is therefore crucial for increasing the diagnosis of infected people, who can then obtain better care and be cured.

## Contributors

Conception and design of the study: ENM, MHAM, ZLC, MRAH; Data collection, data analysis and interpretation: ENM, MHAM, HKC, FY, NAA; Drafting of manuscript and critical revision: ENM, MHAM, ZLC, HKC, FY, NAA, MRAH; Approval of the final version of the article: ENM, MHAM, ZLC, HKC, FY, NAA, MRAH.

## Data sharing statement

The dataset used and/or analysed are included in this published article and supplementary material. Also available from the corresponding author upon reasonable request.

## Declaration of interests

All authors disclose that there is no competing interest. All authors had no potential conflict of interest regarding the publication of this article.
