# Supplementary material for: Hepatitis C virus (HCV) infection in Malaysia: findings from a nationwide cross-sectional study
Source: Lancet Reg Health West Pac. 2023 May 25;36:100802. doi: 10.1016/j.lanwpc.2023.100802 (PMC10220398; doi:10.1016/j.lanwpc.2023.100802)
Supplement: Supplementary material [file mmc1.docx]

**Supplementary Materials**

| **Table contents** | | **Page** |
| --- | --- | --- |
| 1.0 | Sample size determination and samples distribution | 2 |
| 2.0 | Data collection | 2 |
| 3.0 | Biospecimen management and laboratory Testing | 3 |
| 4.0 | Covid-19 prevention during data collection | 3 |
| 5.0 | Data analysis | 3 |
| 6.0 | Limitation of the study | 4 |
| 7.0 | Data of new HCV chronic infection, HCV notification rate and HCV treatment | 4 |
| 8.0 | References | 4 |

1. **Sample size determination and samples distribution**

The sample size was calculated according to the largest prevalence of Hepatitis C in Malaysia (2·5%),^1^ Margin of error (e), and 95% confidence interval. A single proportion formula for the estimation of prevalence:

N_SRS_ ≥ [ Z^2^_α/2_ P(1-P) / e^2^ ]

Few adjustments were made to ensure an optimum sample size:

1. Adjusted for a finite population (Based on 2020 projected population)

*n* ≥ [ *n_S_*_RS_ ] / [ 1+ (*n*_SRS_ / N) ]

b. Adjusted for the design effect (deff), where n(complex)= n(srs)*deff

c. Adjusted the n(complex) considering expected non-response rates of 50%, considering for blood taking via venepuncture procedure, n(adj)=n(complex)* (1+non-response rate).

A complex sampling with two-stage stratified involved; sampling of enumeration blocks (EBs), and the sampling of non-institutional living quarters (LQ) within the EBs. Twenty LQs were chosen at random from each of the selected EBs. All eligibles households (aged 15 years and older) within the LQs were selected for this study. A total 2,260 LQs were chosen from 113 EBs with an estimated 5,000 people will participate in this study.

**Table 1S: Distribution of Enumeration Blocks (EBs) based on the area for data collection**

| **Zone** | **Enumeration Block (EB)** | | |
| --- | --- | --- | --- |
|  | **Strata** | | **Total** |
|  | **Urban area** | **Rural area** |  |
| South  (Including Johor, Melaka and Negeri Sembilan state) | 11 | 9 | 20 |
| Central  (Including Selangor State, Wilayah Persekutuan Kuala Lumpur dan Wilayah Persekutuan Putrajaya) | 19 | 4 | 23 |
| North  (Including Perak, Pulau Pinang, Kedah, and Perlis state) | 10 | 10 | 20 |
| East Coast  (Including Kelantan, Terengganu, and Pahang state) | 11 | 9 | 20 |
| Borneo  (Including Sabah & Labuan and Sarawak state) | 14 | 16 | 30 |
| **Total** | **65** | **48** | **113** |

Note: the EB allocated to each state was based on the population size for each state.

**2.0 Data collection**

A data collection was conducted between 7^th^ August until 11^th^ October 2020 involved all states in Malaysia. Written consents and assent (participants under 18 years old) were obtained from all the participants prior to data collection. Two teams were allocated for each zone during the data collection. Each team consists of two research assistants and one medical practitioner. Face-to-face interviews were conducted by trained research assistants at the respondent’s house, for sociodemographic data, and HCV status. The interview was done using the mobile tables device and the data were uploaded to the NIH’s Survey Creation System (SCS) server. All questions were adopted from the NHANES study which were translated and back-translated before being used.^2^ The interviewer showed the respondents the medicine picture of the HCV medication used in Malaysia or will check their medication card to ensure that respondents are aware that the therapy they received was for HCV. For the blood collection process, respondents were instructed to attend the data collection center which was held in the canopy (equipped with power supply) rented by the data collection team, or at the public hall, depending on what was appropriate in their various enumeration block (EB) locations. The Living Quarters (LQs) was marked as non-responsive if it had been visited for three times but no one was home, empty house and respondents refused to participated.

**3.0 Biospecimen management and laboratory Testing**

Blood samples were drawn into yellow Gel Tube via venepuncture by trained medical personnel amounting to 5·0 ml (for HCV test) from the all respondents. After blood collection, the blood sample was allowed to clot for approximately 30 minutes by leaving disturbing under the room temperature before the centrifugation procedure. The blood tube was centrifuged on-site (same place with the blood sample collection was done using the portable centrifuge) to ensure the blood stability. Sample were store into the portable chiller before delivered to the accrediting laboratory at end of data collection on those days or less than 48 hours after blood collection was done. In order to ensure the quality of the blood before it was submitted for testing, a thermologger was used to monitor the temperature in the portable chiller, which was kept between 2⁰C and 8⁰C. Blood samples were sent to the accrediting laboratory, and tested for HCV antibodies (anti-HCV) and HCV core antigen (anti-HCV core).^3^

The Architect Anti-HCV assay, a chemiliminescent microparticle immunoassay (CMIA) for the qualitative detection of antibody in human serum, was used to screen all the respondents blood samples for Hepatitis C antibodies (Anti-HCV). Hepatitis C core antigen (HCV core Ag) testing was done on samples that tested positive for anti-HCV. HCV core Ag was done using Architect HCV Ag assay, a CMIA for the quantitative determination of core antigen to hepatitis C in human serum. The laboratory procedure as in product package insert.^3,4^ The interpretation of serological tests was done according to the CDC guideline.^5^

All the Anti-HCV and HCV Core Ag results was validated by the pathologist or medical officer. Phone calls were made to respondents who tested positive and advised of their HCV status. The authorized medical officer gave the referral letter and instructed them to attend to the nearest government facility for further assessments. Results for the negative tests were mailed to the respondents.

**4.0 Covid-19 prevention during data collection**

In order to control the spread of Covid-19 during data collection, preventive measures have been taken strictly for all data collection team involved. All team are informed and always reminded about covid-19 infection information. They were explained in depth about this infection to ensure the transmission of this disease does not occur. All team members must perform antigen Covid 19 tests (Nasopharyngeal swab rapid kits) occasionally every two weeks in addition to wearing full PPE (face masks, gloves, grown, face shields, etc.) and practicing social distancing by making sure that all routes are one-way. To minimize traffic at the blood collection center, respondents who came for blood collection were assigned an appointment. Team members who develop symptoms of COVID-19 or tested positive for Covid-19 will be given time off and not required for data collection activity.

**5.0 Data analysis**

To obtain population estimates, descriptive data and a complex sample analysis were done with weight factors including design weight, non-response rate, and post-stratification weight and adjusted for the Malaysian population projections. SPSS Version 26·0 was used to analyse the data. The prevalence was reported with 95% confidence intervals.

The following formula was used to estimate the samples weight:

W_final_ = W_1_ × W_2_ × F × PS

W_1_ = the inverse of probability of selecting the EBs

W_2_ = the inverse probability of selecting the LQ within selected EB

F = the non-response adjustment factor for individual and LQ

PS = a post-stratification adjustment factor calculated by gender, strata, age, and ethnicity

**6.0 Limitation of the study**

This was a cross-sectional study conducted during the peak of the COVID-19 pandemic in 2020. The response rate was decreased because some people were hesitant to communicate with strangers, and some decided not to leave their homes for blood collection. Hence, the sample size was enough to describe the national prevalence only (not enough sample for prevalence by zone or strata). The data were inadequate for further stratification analysis, which incurs very high RSEs. However, this study is the first to look at HCV seroprevalence across the whole country, which is important for future efforts to prevent HCV in the community. As this is a population study i.e data collection done in LQs excluding institutional LQs such as prisons and drug rehabilitation centres, the key risk groups such as people who inject drugs or prisoners was not estimated in the prevalence.

1. **Data of new HCV chronic infection, HCV notification rate and HCV treatment.**

**Table S2: New chronic HCV infection and notification rate, Malaysia (2018-2022)**

|  | **2018** | **2019** | **2020** | **2021** | **2022** |
| --- | --- | --- | --- | --- | --- |
| New chronic infection | 2,842 | 3,438 | 3,300 | 2,804 | 4,626 |
| Notification rate  (Per 100,000 population) | 8·77 | 10·55 | 10·10 | 8·59 | 14·17 |

Source: Disease Control Division, Ministry of Health Malaysia.

**Table S3: Total HCV patients were treated using Direct-Acting Antivirals (DAAs) in Malaysia (2018-2022)**

|  | **2018** | **2019** | **2020** | **2021** | **2022** |
| --- | --- | --- | --- | --- | --- |
| DAAs | 1168 | 2970 | 3883 | 4217 | 4819 |

Note: HCV treatment using DAA in Malaysia was started in 2018.

Source: Pharmacy Services Program, Ministry of Health Malaysia.

**8.0 References**

1. Mcdonald SA, Mohamed R, Dahlui M, Naning H, Kamarulzaman A. Bridging the data gaps in the epidemiology of hepatitis C virus infection in Malaysia using multi-parameter evidence synthesis. BMC Infect Dis. 2014; **14:** 564.
2. Center for Health Statistics N. NHANES 2015-2016 Hepatitis Questionnaire. 2015. Accessed February 3, 2023. https://wwwn.cdc.gov/nchs/data/nhanes/2015-2016/questionnaires/HEQ_I.pdf
3. Anti-HCV product package insert. <https://www.ilexmedical.com/files/PDF/AntiHCV_ARC.pdf> Accessed February 5, 2023.
4. HCV core antigen product package insert. <https://www.ilexmedical.com/files/PDF/HCVAg_ARC.pdf> Accessed February 5, 2023.
5. Center for Disease Control and Prevention. Interpretation of Results of Tests for HCV Infection | CDC. Accessed February 3, 2023. https://www.cdc.gov/hepatitis/hcv/HCVTestResults-InterpretationAndActions.htm
